# Longitudinal adoption of robotic surgery and changes in prostate cancer care delivery: a 10-year institutional case study

**DOI:** 10.3389/frhs.2026.1836470

**Published:** 2026-07-24

**Authors:** Min-Che Tung, Lihua Huang, Yi-Sheng Lin, Chao-Yu Hsu, Wei-Chun Weng, Lonfye Lye, Yen-Chuan Ou

**Affiliations:** Department of Surgery, Tungs’ Taichung MetroHarbor Hospital, Wuqi, Taichung, Taiwan

**Keywords:** care pathways, health systems, integrated care, medical technology adoption, prostate cancer, robotic surgery

## Abstract

High-cost medical technologies are usually evaluated by clinical outcomes and cost-effectiveness, while their organizational effects are less often examined. We conducted a 10-year longitudinal institutional case study (2012–2022) of 395 prostate cancer procedures, combining process tracing, descriptive quantitative trends, implementation records, and workflow documentation. Three quantitative indicators were examined across four adoption stages: procedural volume, biopsy-free surgery, and final pathological confirmation. Biopsy-free surgery increased from 13 of 55 procedures (23.6%) during experimentation to 96 of 125 (76.8%) during maturity, and final pathological confirmation increased from 22 of 55 (40.0%) to 96 of 125 (76.8%). Cochran–Armitage tests showed significant increasing trends for both indicators (Z = 6.48 and 5.25, respectively; both *p* < 0.001). Institutional records documented concurrent introduction of training, standardized protocols, structured assessment, multidisciplinary processes, and follow-up pathways. These findings demonstrate temporal changes in clinical practice and suggest, without quantitatively establishing, a broader evolution toward more coordinated prostate cancer care. Evaluation of high-cost technologies should therefore distinguish measured clinical changes from organizational interpretations based on implementation evidence.

## 1 Introduction

### 1.1 From technology adoption to health service transformation

#### 1.1.1 Theoretical perspectives on health technology adoption

The adoption of medical technologies can be understood through several complementary theoretical perspectives. Innovation diffusion theory emphasizes that new technologies are not adopted instantly, but spread through stages influenced by perceived advantage, compatibility with existing practice, professional acceptance, organizational readiness, and routinization (1). From this perspective, robotic-assisted surgery adoption can be viewed as a progressive process in which early experimentation is followed by broader use, standardization, and eventual integration into routine care.

Research on innovation in service organizations further suggests that technology adoption is shaped not only by the technical characteristics of the innovation, but also by organizational context, implementation processes, professional networks, and system readiness (2). In healthcare settings, complex technologies often require changes in work routines, coordination across professional groups, resource allocation, and governance structures. Therefore, successful adoption depends not only on clinical performance, but also on how the technology becomes embedded within institutional workflows and care delivery systems.

A complementary platform and infrastructure perspective highlights that some technologies may become organizational infrastructures rather than isolated procedural tools. Platform theory suggests that technologies can create value by connecting different users, processes, and activities within a system (3–5). Applied to healthcare, this perspective suggests that robotic-assisted surgery may influence not only the operative procedure, but also the organization of diagnostic evaluation, surgical planning, perioperative management, rehabilitation, and follow-up. This theoretical framing supports the present study's focus on robotic-assisted surgery adoption as a longitudinal process of service organization and care pathway integration. In addition, the NASSS framework highlights that the adoption, scale-up, spread, and sustainability of health technologies are influenced by interactions among the clinical condition, technology, value proposition, users, organization, wider system, and long-term embedding processes (6).

High-cost medical technologies can influence not only clinical practice but also the organization of care. Their implementation often requires substantial investment, specialized workforce coordination, workflow adaptation, and institutional governance (1, 2, 7, 8). However, these organizational effects are less frequently evaluated than clinical outcomes and cost-effectiveness.

Robot-assisted surgery illustrates this issue. Although its clinical outcomes have been widely studied (9–11), less is known about how adoption unfolds within institutional care pathways. Table 1 summarizes general perioperative and functional outcome patterns reported in prior comparative studies and provides clinical context for the present case study.

**Table 1:** General perioperative and functional outcome patterns reported for open, laparoscopic, and robot-assisted radical prostatectomy.

Outcome domain	Outcome measure	Open radical prostatectomy	Laparoscopic radical prostatectomy	Robot-assisted radical prostatectomy
Perioperative outcomes	Estimated blood loss	Highest	Intermediate	Lowest
	Blood transfusion requirement	Highest	Lower	Lowest
Postoperative recovery	Postoperative pain	Greater	Intermediate	Less
	Length of hospital stay	Longer	Intermediate	Shorter
	Catheterization duration	Longer	Intermediate	Shorter
Postoperative safety	Perioperative morbidity/postoperative complications	Higher	Intermediate	Lower
Functional recovery	Return to normal activity	Slower	Intermediate	Faster
	Urinary continence recovery	Less favorable	Intermediate	More favorable
	Sexual function recovery*	Less favorable	Intermediate	More favorable

This table summarizes general comparative patterns reported in previous studies and meta-analyses. It is provided as clinical background and does not represent original findings from the present institutional dataset. *Among patients with normal preoperative erectile function. Reported outcomes may vary according to patient selection, surgeon experience, outcome definitions, and follow-up duration.

#### 1.1.2 Technology as a platform for integrated care

While medical technologies are often introduced as tools to improve specific clinical procedures, their impact frequently extends beyond individual interventions. In practice, high-cost technologies can act as platforms that connect different stages of care, linking diagnosis, treatment, and follow-up into a more coordinated system (3, 4).

From a health services perspective, this shift is important because it changes how resources are used and how decisions are made. Technologies that function as platforms may create new demands for coordination, data integration, and team-based care (12, 13).

#### 1.1.3 Policy context, screening, and diagnostic uncertainty

Prostate cancer provides a useful example, as it combines increasing disease burden with ongoing challenges in diagnosis and treatment decision-making. While earlier detection through screening can improve outcomes (14–16), it also introduces uncertainty, including risks of overdiagnosis and overtreatment (17).

In Taiwan, prostate cancer incidence has risen steadily, influenced by population aging and expanded use of PSA testing (18, 19). These clinical challenges are closely linked to the organization of the health system, where policy frameworks and institutional incentives shape technology adoption and use.

**Figure 1:**
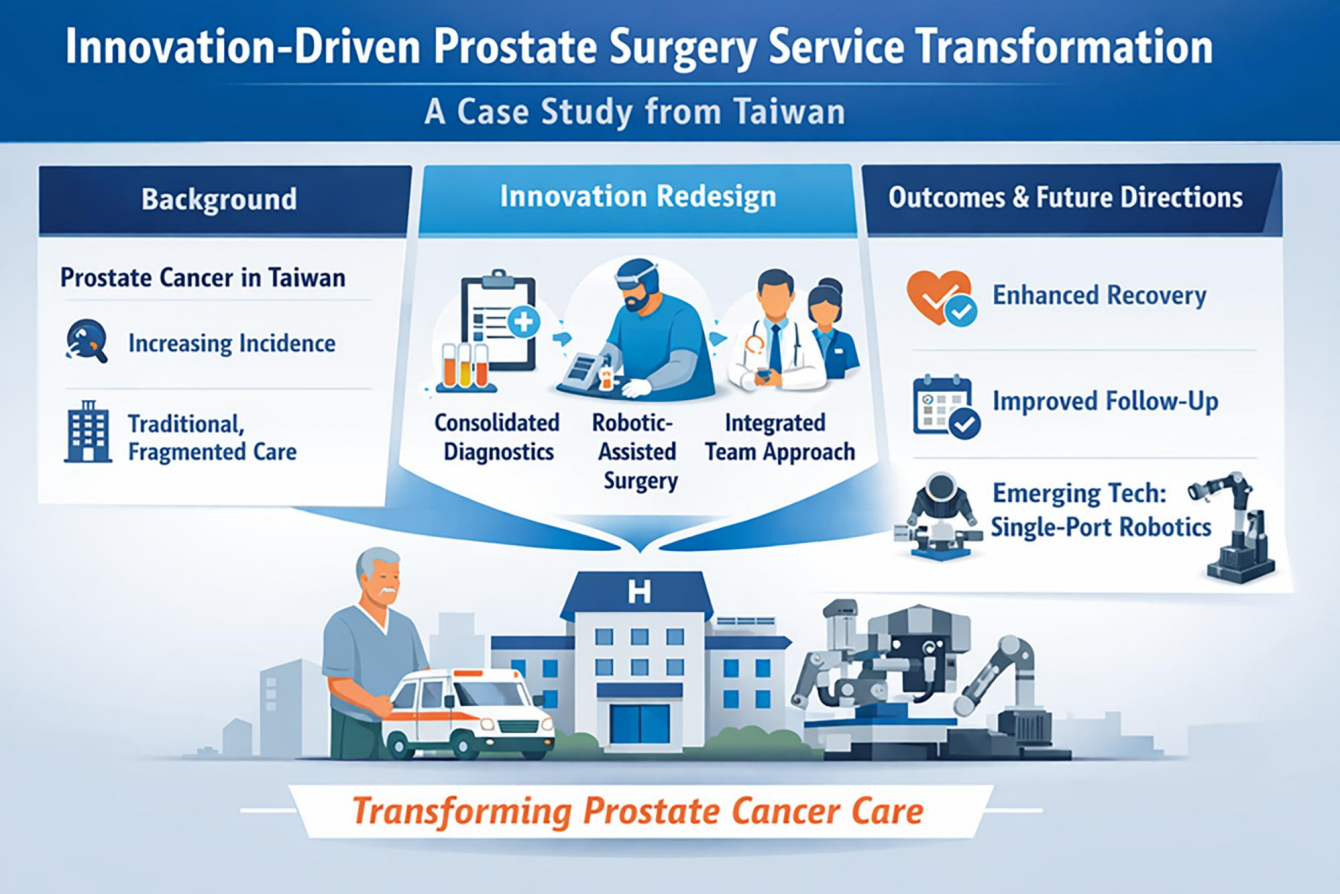
Conceptual diagnostic and treatment pathway for therapeutic diagnostic radical prostatectomy.

Figure 1 provides a conceptual overview of the diagnostic and treatment pathway for therapeutic diagnostic radical prostatectomy. It illustrates how PSA assessment, multiparametric magnetic resonance imaging, structured risk prediction, and surgical management may be integrated when managing diagnostic uncertainty in prostate cancer care (14, 17, 20). Supplementary Table S1 summarizes the variables included in the structured preoperative risk prediction nomogram (21) and provides context for how clinical, imaging, laboratory, and pathology-related information were incorporated into diagnostic and treatment decision-making.

### 1.2 Study design

This 10-year longitudinal institutional case study examined robotic-assisted prostate cancer surgery from 2012 to 2022. The analysis focused on technology implementation, documented workflow changes, multidisciplinary processes, and temporal patterns in clinical decision-making.

A longitudinal design was used because adoption occurred progressively rather than as a single implementation event. The study therefore examined four analytically defined stages: experimentation, expansion, stabilization, and maturity.

Implementation was examined using institutional records of system introduction, surgeon training and credentialing, protocol development, operating-room resource allocation, and expansion of patient eligibility. Workflow integration was examined using care pathways, preoperative assessment procedures, multidisciplinary processes, perioperative protocols, follow-up schedules, and coordination records across clinical services.

Clinical practice was assessed using three quantitative indicators available across all stages: procedural volume, biopsy-free surgery, and final pathological confirmation. Organizational observations were derived from implementation records and workflow documentation and were analyzed separately from the quantitative indicators.

This design allowed quantitative clinical trends to be interpreted alongside documented institutional developments without treating the latter as objectively measured organizational outcomes.

### 1.3 Study setting

The study was conducted within Taiwan's healthcare system, which is characterized by universal coverage under a single-payer National Health Insurance program. At the same time, hospitals operate in a competitive service environment, and high-cost technologies such as robotic-assisted surgery may not be fully covered by routine reimbursement. This creates a context in which decisions about adopting and utilizing new technologies are shaped by both national policy, patient demand, institutional investment capacity, and hospital-level service differentiation.

The analysis focused on a tertiary referral hospital with extensive experience in robotic-assisted prostate cancer surgery. This institution was selected because it had implemented the technology early and accumulated sufficient experience to allow examination of the entire adoption process, from initial experimentation to stable routine use. This setting provided a suitable context for understanding how technology integration can influence care delivery over time.

### 1.4 Data sources

The analysis drew on multiple sources of institutional data covering the period from 2012 to 2022. These included aggregated records of 395 prostate cancer procedures, defined as patients who underwent prostate cancer surgery at the study institution during the study period, primarily robot-assisted radical prostatectomy. Annual procedural volumes were reviewed to examine changes in technology utilization over time, including the number of robotic prostate cancer surgeries performed each year and changes in case volume across the different adoption phases.

Indicators related to diagnostic and pathological outcomes were also examined. These included whether surgery was performed with or without prior biopsy confirmation, whether prostate cancer was confirmed on final surgical pathology, and the degree of alignment between preoperative clinical assessment and postoperative pathological findings. These indicators were used to assess how diagnostic decision-making evolved as robotic-assisted surgery became more established in routine care.

Institutional implementation records were reviewed to understand how the robotic surgical technology was introduced and scaled within the hospital. Examples of these records included documentation related to the initial introduction of the robotic surgical system, surgeon training and credentialing, development of operative protocols, allocation of operating room resources, and gradual expansion of eligible patient groups over time. These records helped clarify how the technology moved from limited early use to broader routine adoption.

Internal workflow documentation was also reviewed to examine how the technology was integrated into clinical practice. Examples included prostate cancer care pathways, preoperative assessment procedures, multidisciplinary discussion processes, perioperative management protocols, postoperative follow-up schedules, and coordination procedures among urology, radiology, pathology, anesthesia, nursing, and rehabilitation teams. These materials provided insight into how diagnostic evaluation, surgical planning, operative management, and follow-up care were reorganized into a more coordinated clinical workflow.

Together, these data sources allowed the study to examine not only changes in procedural volume and pathological outcomes, but also the organizational processes through which robotic-assisted prostate cancer surgery was introduced, scaled, and embedded into routine prostate cancer care.

### 1.5 Data analysis

Process tracing was used to reconstruct the sequence of robotic surgery adoption, identify implementation milestones, and compare documented workflow developments with quantitative trends over time.

First, a chronological implementation timeline was constructed from annual procedural volumes, implementation records, and workflow documents. Milestones included system introduction, training and credentialing, protocol development, structured risk assessment, and follow-up pathway implementation.

Second, transition points were identified from changes in utilization, patient eligibility, workflow documentation, and diagnostic practice. These transition points informed the four adoption stages.

The four stages were defined using chronological implementation milestones, procedural volume trends, and documented workflow developments rather than a single volume threshold or policy event. Experimentation (2012–2015) reflected limited early use and selective case inclusion; expansion (2015–2018) reflected broader utilization and eligibility; stabilization (2018–2020) reflected increasing protocolization and structured assessment; and maturity (2020–2022) reflected routine institutional use within established pathways.

The stages were used as an interpretive longitudinal framework. They were not treated as independently validated measures of organizational performance or as evidence that robotic surgery caused subsequent workflow changes.

Within each stage, organizational observations were examined in three domains: care delivery, multidisciplinary coordination, and diagnostic and treatment decision-making. These domains were assessed from institutional documents, whereas biopsy status and final pathological confirmation were analyzed quantitatively.

For quantitative analysis, stage-specific proportions were calculated using the total number of procedures in each stage as the denominator. Cochran–Armitage tests assessed trends in biopsy-free surgery and final pathological confirmation across the ordered stages. Statistical significance was defined as *p* < 0.05.

The three quantitative indicators were procedural volume, biopsy-free surgery rate, and final pathological confirmation rate. Procedural volume described utilization, whereas the latter two characterized temporal changes in diagnostic and treatment practice.

### 1.6 Qualitative analysis

Qualitative analysis was conducted using institutional implementation records and internal workflow documentation. These materials were reviewed by members of the study team with experience in prostate cancer care, robotic surgery implementation, and health services research. The purpose of the review was to identify how robotic-assisted surgery was introduced, scaled, and integrated into prostate cancer care delivery over time.

The analysis followed a structured qualitative content analysis approach. First, all documents were organized chronologically according to the four adoption stages identified in the study: experimentation, expansion, stabilization, and maturity. This allowed the analysis to examine how organizational changes developed sequentially rather than treating technology adoption as a single implementation event.

Second, documents were reviewed using predefined analytical domains derived from the study objectives and conceptual approach. These domains included technology implementation, care pathway redesign, multidisciplinary coordination, workflow standardization, and diagnostic and treatment decision-making. Examples of reviewed materials included documentation on surgeon training and credentialing, operative protocol development, operating room resource allocation, preoperative assessment procedures, multidisciplinary discussion processes, perioperative management protocols, postoperative follow-up schedules, and coordination among urology, radiology, pathology, anesthesia, nursing, and rehabilitation teams.

Third, recurring themes within these domains were identified and compared across adoption stages. Particular attention was given to whether care organization shifted from a fragmented, department-based model toward a more integrated pathway linking diagnosis, surgery, perioperative care, rehabilitation, and follow-up. The analysis was not based on a formal Consolidated Framework for Implementation Research coding structure. Instead, the analytical categories were developed from the study aims, the longitudinal adoption framework, and recurring patterns observed in the institutional documents.

Finally, qualitative findings were triangulated with descriptive quantitative indicators, including annual procedural volumes, biopsy-free surgery rates, and final pathological confirmation rates. This triangulation helped connect documented organizational changes with observed changes in clinical practice and supported interpretation of how robotic-assisted surgery became embedded into routine prostate cancer care.

### 1.7 Conceptual approach

The analysis was informed by an interdisciplinary perspective drawing on innovation diffusion theory, research on innovation in service organizations, and platform/infrastructure theory. Innovation diffusion theory guided the interpretation of robotic-assisted surgery adoption as a staged process involving experimentation, expansion, stabilization, and routinization (1). Service organization theory informed the analysis of how adoption was associated with changes in workflows, professional coordination, organizational routines, and governance (2, 22). Platform and infrastructure perspectives supported the interpretation of robotic-assisted surgery as a technology-enabled organizational platform that may connect multiple stages of care rather than functioning only as a discrete surgical tool (3–5). In this study, the robotic surgical technology was understood not only as a clinical tool, but also as a technology-enabled organizational platform. A platform was defined as an infrastructure that extends beyond a single surgical procedure and connects multiple stages of care, including diagnostic assessment, treatment planning, surgery, perioperative management, rehabilitation, and follow-up.

To operationalize this conceptual approach, three key domains were linked to observable indicators. First, coordination was assessed through evidence of multidisciplinary involvement and documented collaboration among urology, radiology, pathology, anesthesia, nursing, and rehabilitation teams. Examples included multidisciplinary case discussions, shared preoperative planning, coordination of perioperative care, and structured postoperative follow-up.

Workflow integration was assessed from the documented presence and development of care pathways, assessment procedures, perioperative protocols, rehabilitation processes, and follow-up schedules.

Clinical decision-making was assessed quantitatively through biopsy status and final pathological confirmation and descriptively through documented use of structured risk assessment.

This approach linked conceptual domains to two evidence types: quantitative procedural indicators and documented institutional observations.

Although established implementation frameworks such as the Consolidated Framework for Implementation Research are relevant to the broader topic of technology implementation, the present study did not apply formal CFIR coding; instead, it used a study-specific analytical framework focused on adoption stage, workflow integration, multidisciplinary coordination, and diagnostic decision-making.

### 1.8 Rigor and trustworthiness

Credibility was supported by use of multiple data sources, chronological comparison across stages, and triangulation of quantitative trends with implementation and workflow documents.

Because formal independent double coding was not performed, qualitative interpretations were treated as descriptive and hypothesis-generating rather than as validated organizational measurements.

## 2 Results

The quantitative analysis focused on three indicators available across all four adoption stages: procedural volume, biopsy-free surgery, and final pathological confirmation. Documented organizational developments are reported separately as implementation milestones and interpreted alongside, rather than as equivalent to, the quantitative findings.

### 2.1 Procedural volume and adoption trends

Procedural volume increased across the four adoption stages, from 55 procedures during experimentation to 98 during expansion, 117 during stabilization, and 125 during maturity (Tables 2, 3). This pattern documents increasing utilization over time but does not by itself demonstrate improved care delivery.

**Table 2:** Documented implementation milestones and corresponding quantitative indicators across robotic surgery adoption stages.

Adoption stage	Period	Documented implementation milestone	Evidence source	Quantitative indicator
Experimentation	2012–2015	Introduction of the robotic surgical program; initial surgeon training and credentialing; selective patient inclusion	Training records, credentialing documents, operative records	55 procedures; biopsy-free surgery: 13/55 (23.6%); pathological confirmation: 22/55 (40.0%)
Expansion	2015–2018	Expansion of robotic surgery use and broader patient eligibility	Operative records, eligibility criteria, implementation records	98 procedures; biopsy-free surgery: 54/98 (55.1%); pathological confirmation: 49/98 (50.0%)
Stabilization	2018–2020	Introduction of standardized perioperative protocols and structured risk assessment	Clinical pathway documents, perioperative protocols, risk-assessment records	117 procedures; biopsy-free surgery: 73/117 (62.4%); pathological confirmation: 71/117 (60.7%)
Maturity	2020–2022	Routine use of structured care pathways, multidisciplinary assessment, and standardized follow-up	Workflow documents, multidisciplinary records, follow-up protocols	125 procedures; biopsy-free surgery: 96/125 (76.8%); pathological confirmation: 96/125 (76.8%)

Implementation milestones were identified from dated institutional records and workflow documents. Quantitative indicators were derived from the 395 procedures included in the study. The table describes the chronological coexistence of implementation changes and clinical practice trends; it does not establish that organizational changes caused the observed quantitative outcomes.

**Table 3:** Trends in biopsy-free surgery and pathological confirmation rates across adoption stages, 2012–2022.

Adoption stage	Study period	Total patients/procedures, N	Biopsy-free surgery, N (%)	Cancer confirmed on final pathology, N (%)
Experimentation	2012–2015	55	13 (23.6%)	22 (40.0%)
Expansion	2015–2018	98	54 (55.1%)	49 (50.0%)
Stabilization	2018–2020	117	73 (62.4%)	71 (60.7%)
Maturity	2020–2022	125	96 (76.8%)	96 (76.8%)
Overall	2012–2022	395	236 (59.7%)	238 (60.3%)

Percentages were calculated using the total number of patients/procedures within each adoption stage as the denominator. Values are presented as N (%). Cochran–Armitage tests for trend were used to assess changes across ordered adoption stages. Significant increasing trends were observed for both biopsy-free surgery rates (Z = 6.48, *p* < 0.001) and final pathological confirmation rates (Z = 5.25, *p* < 0.001). Stage definitions were based on institutional implementation milestones, procedural volume trends, and workflow documentation rather than a single volume threshold or national policy change.

### 2.2 Documented implementation milestones

Institutional records documented a sequence of implementation activities, including system introduction, surgeon training and credentialing, expansion of patient eligibility, introduction of standardized perioperative protocols and structured assessment, and establishment of follow-up pathways.

Table 2 presents these milestones together with the corresponding procedural volume, biopsy-free surgery rate, and final pathological confirmation rate for each stage.

The implementation milestones and quantitative trends occurred during the same periods; however, the study design does not establish that the organizational developments caused the observed clinical changes.

### 2.3 Changes in diagnostic decision-making

Biopsy-free surgery increased across the adoption stages, from 13 of 55 procedures (23.6%) during experimentation to 96 of 125 (76.8%) during maturity.

Final pathological confirmation increased from 22 of 55 procedures (40.0%) during experimentation to 96 of 125 (76.8%) during maturity. Cochran–Armitage tests showed significant increasing trends for biopsy-free surgery (Z = 6.48, *p* < 0.001) and final pathological confirmation (Z = 5.25, *p* < 0.001) (Table 3).

In the maturity stage, both rates were 76.8%. This numerical alignment indicates temporal concordance between the two aggregated indicators but should not be interpreted as proof of improved diagnostic accuracy or causality.

The observed trends occurred alongside documented use of structured risk assessment incorporating clinical, imaging, laboratory, and pathology-related variables (Supplementary Table S1).

## 3 Discussion

### 3.1 From clinical innovation to service transformation

The study identified significant temporal changes in biopsy-free surgery and final pathological confirmation across four stages of robotic surgery adoption. Institutional records also documented concurrent developments in training, protocols, structured assessment, multidisciplinary processes, and follow-up pathways. These two evidence streams should be interpreted together but not treated as equivalent.

The findings suggest that technology adoption occurred alongside organizational adaptation. However, the available data do not quantify the degree or quality of care integration, coordination, workflow performance, service efficiency, or patient-centeredness, and they do not establish causality.

### 3.2 Integration of care and organizational change

Institutional documentation described a transition from largely separate service components toward more structured pathways linking assessment, surgery, perioperative care, and follow-up. This represents a documented organizational pattern rather than a quantitatively measured improvement.

The pattern is consistent with the view that the organizational value of complex technologies depends on how institutions adapt workflows and coordination mechanisms around them (2, 22).

### 3.3 Improving decision-making under uncertainty

The increases in biopsy-free surgery and final pathological confirmation indicate changing diagnostic and treatment practices over time. Their concurrent rise suggests greater temporal concordance between preoperative decision-making and postoperative pathology, but aggregated data cannot determine diagnostic accuracy at the patient level.

These trends coincided with documented use of structured risk assessment. Because the study was descriptive and observational, it cannot determine whether robotic surgery or any specific organizational change directly improved decision-making.

The findings nevertheless support further evaluation of decision-support processes, multidisciplinary assessment, and standardized pathways using patient-level and comparative designs.

### 3.4 Learning, adaptation, and use of technology

The four stages of experimentation, expansion, stabilization, and maturity provide an interpretive framework for the longitudinal institutional trajectory. They were derived from documented milestones and procedural trends rather than from validated organizational scales.

Table 4 summarizes this framework. The available quantitative indicators do not directly measure care integration, coordination quality, workflow standardization, service efficiency, or patient-centered care; therefore, the stage-specific organizational descriptions should be interpreted as contextual evidence.

**Table 4:** Interpretive framework of robotic surgery adoption across the longitudinal institutional case.

Adoption stage	Period	Documented institutional context	Quantitative procedural indicator	Analytical stage description
Experimentation	2012–2015	Initial implementation, surgeon training and credentialing, selective case inclusion	55 procedures; biopsy-free surgery: 13/55 (23.6%); pathological confirmation: 22/55 (40.0%)	Early implementation and limited use
Expansion	2015–2018	Broader use of robotic surgery and expansion of eligible patients	98 procedures; biopsy-free surgery: 54/98 (55.1%); pathological confirmation: 49/98 (50.0%)	Broader use and service expansion
Stabilization	2018–2020	Introduction of standardized perioperative protocols and structured assessment processes	117 procedures; biopsy-free surgery: 73/117 (62.4%); pathological confirmation: 71/117 (60.7%)	Greater standardization of clinical processes
Maturity	2020–2022	Routine use of the robotic surgical program within established care pathways	125 procedures; biopsy-free surgery: 96/125 (76.8%); pathological confirmation: 96/125 (76.8%)	Routine use within established care pathways

This framework represents the authors' interpretation of the longitudinal institutional case and should not be interpreted as a quantitatively validated measure of organizational performance or as evidence of a causal effect.

Observed changes may also reflect institutional learning, evolving diagnostic practices, increased mpMRI use, guideline changes, patient selection, and reimbursement conditions. Robotic surgery adoption should therefore be understood as one component of a broader institutional process rather than as the sole cause of change.

### 3.5 Practical implications for health services and policy

For healthcare organizations, adoption of high-cost technologies should be accompanied by explicit workflow design, multidisciplinary governance, and ongoing evaluation. Importantly, organizational performance should be assessed using measurable indicators rather than inferred solely from implementation documents.

For policy-makers, evaluation should consider both clinical outcomes and how technologies are embedded within care pathways. Future studies should quantify pathway adherence, waiting times, multidisciplinary review, follow-up completion, complications, and patient-reported outcomes.

These considerations are also relevant to other complex technologies, including artificial intelligence and digital platforms, which require coordination, data governance, and clear accountability (23–25).

Taiwan's single-payer National Health Insurance system may facilitate continuity across diagnosis, treatment, and follow-up, while hospital competition and incomplete reimbursement for high-cost technologies may shape institutional adoption decisions.

Accordingly, the observed trajectory may not generalize to fragmented financing systems or institutions with different incentives and capacities for workflow redesign.

### 3.6 Clinical implications

Clinically, the quantitative findings document changing use of biopsy-free surgery and final pathological confirmation over time. They do not demonstrate improvements in hospital stay, complications, functional recovery, treatment delay, continuity, or patient experience.

The documented use of structured assessment and multidisciplinary processes supports the rationale for coordinated decision-making, but their clinical effectiveness requires direct patient-level evaluation.

Patient-level factors such as health literacy, socioeconomic circumstances, travel burden, family support, and follow-up capacity may influence participation in pathway-based care. These factors were not measured in the present study and should be considered in future evaluations.

### 3.7 Future applications and policy implications

Future research should test whether documented pathway developments translate into measurable improvements in diagnostic accuracy, treatment timeliness, complications, follow-up completion, patient experience, and resource use.

Such studies should use patient-level data, explicit comparator groups, and validated measures of integration and coordination.

### 3.8 Limitations

This study has several limitations. First, it was a single-institution retrospective case study, limiting generalizability. Second, the analysis used aggregated rather than patient-level data and therefore could not assess individual diagnostic accuracy, clinical outcomes, or patient experience.

Third, the observational design cannot establish causal relationships between robotic surgery adoption, organizational developments, and clinical trends. Other influences include institutional learning, evolving guidelines, mpMRI use, patient selection, and reimbursement conditions.

Fourth, organizational changes were identified from implementation and workflow documents rather than validated quantitative measures of integration, coordination, pathway adherence, efficiency, or patient-centered care.

Fifth, formal independent double coding and inter-coder reliability assessment were not performed, so the qualitative synthesis may be subject to researcher interpretation.

Finally, stage boundaries were analytically defined from institutional milestones and procedural trends and were not independently validated.

Despite these limitations, the study provides a longitudinal account of quantitative clinical trends alongside documented institutional developments during robotic surgery adoption.

The findings are most applicable to institutions with sufficient capacity to implement complex technologies and redesign related workflows. Transferability to other financing and organizational settings should be assessed empirically.

## 4 Conclusion

Across the 10-year adoption period, robot-assisted prostatectomy use was accompanied by significant increases in biopsy-free surgery and final pathological confirmation, together with documented implementation of training, structured workflows, multidisciplinary processes, and follow-up pathways.

The quantitative findings demonstrate temporal changes in clinical practice. Institutional records suggest, but do not quantitatively establish, a broader evolution toward more coordinated prostate cancer care.

These findings support evaluation frameworks that distinguish measured clinical outcomes from organizational interpretations and that incorporate direct indicators of integration, coordination, efficiency, and patient experience.

## Data Availability

The raw data supporting the conclusions of this article will be made available by the authors, without undue reservation.
