# Supplementary material for: Longitudinal adoption of robotic surgery and changes in prostate cancer care delivery: a 10-year institutional case study
Source: Front Health Serv. 2026 Jul 24;6:1836470. doi: 10.3389/frhs.2026.1836470 (PMC13447359; doi:10.3389/frhs.2026.1836470)
Supplement: Supplementary file 1 [file Table1.docx]

**Supplementary Table 1. Variables included in the prostate cancer risk prediction nomogram.** Clinical, imaging, laboratory, and pathological variables incorporated into structured preoperative risk estimation

| **Domain** | **Variable** | **Description** |
| --- | --- | --- |
| Demographic | Age | Patient age at evaluation |
| Imaging | Suspicious MRI lesion | Presence of suspicious lesion on mpMRI |
| Imaging | MRI grade | MRI-based lesion grading |
| Laboratory | PSA density (PSAD) | Serum PSA adjusted for prostate volume |
| Laboratory | PSA velocity (PSAV) | Rate of PSA increase over time |
| Clinical | Urinary retention | History or presence of urinary retention |
| Pathology (if available) | Biopsy pathology | Benign, premalignant (HGPIN/ASAP), or malignant |
| Output | Predicted cancer probability | Estimated probability of prostate cancer |
